# Protective Effects of MicroRNA‐200b‐3p Encapsulated by Mesenchymal Stem Cells–Secreted Extracellular Vesicles in Myocardial Infarction Via Regulating BCL2L11

**DOI:** 10.1161/JAHA.121.024330

**Published:** 2022-06-14

**Authors:** Jun Wan, Shaoyan Lin, Zhuo Yu, Zhengkun Song, Xuefeng Lin, Rongning Xu, Songlin Du

**Affiliations:** ^1^ Department of Cardiovascular Surgery Nanfang Hospital Southern Medical University Guangzhou Guangdong China

**Keywords:** apoptosis, Bcl‐2–like protein 11, extracellular vesicles, inflammation, microRNA‐200b‐3p, myocardial infarction, NLR family pyrin domain containing 1, Myocardial Infarction, Myocardial Biology

## Abstract

**Background:**

Extracellular vesicles (EVs) are a popular treatment candidate for myocardial injury. This work investigated the effects of mesenchymal stem cells (MSCs)–secreted EVs–derived miR‐200b‐3p on cardiomyocyte apoptosis and inflammatory response after myocardial infarction (MI) through targeting BCL2L11 (Bcl‐2–like protein 11) .

**Methods and Results:**

EVs from MSCs were isolated and identified. EVs from MSCs with transfection of miR‐200b‐3p for overexpression were injected into MI mice. The effect of miR‐200b‐3p on cardiac function, infarction area, myocardial fibrosis, cardiomyocyte apoptosis, and inflammatory response was determined in MI mice. The targeting relationship between miR‐200b‐3p and BCL2L11 was verified, and the interaction between BCL2L11 and NLR family pyrin domain containing 1 (NLRP1) was also verified. MI mice were injected with an overexpressing BCL2L11 lentiviral vector to clarify whether BCL2L11 can regulate the effect of miR‐200b‐3p on MI mice. EVs from MSCs were successfully extracted. MSCs‐EVs improved cardiac function and reduced infarction area, apoptosis of cardiomyocytes, myocardial fibrosis, and inflammation in MI mice. Upregulation of miR‐200b‐3p further enhanced the effects of MSCs‐EVs on the myocardial injury of MI mice. BCL2L11 was targeted by miR‐200b‐3p and bound to NLRP1. Upregulation of BCL2L11 negated the role of miR‐200b‐3p–modified MSCs‐EVs in MI mice.

**Conclusions:**

A summary was obtained that miR‐200b‐3p–encapsulated MSCs‐EVs protect against MI‐induced apoptosis of cardiomyocytes and inflammation via suppressing BCL2L11.

**Nonstandard Abbreviations and Acronyms**
BCL2L11: Bcl‐2–like protein 11
EV: extracellular vesicle
LVEDD: left ventricular end‐diastolic diameter
LVESD: left ventricular end‐stage systole diameter
LVFS: left ventricular fractional shortening
MI/RI: myocardial ischemia/reperfusion
miRNA: microRNA
MSC: mesenchymal stem cell
NC: negative control
NLRP1: NLR family pyrin domain containing 1
ROS: reactive oxygen species
SOD: superoxide dismutase

**Clinical Perspective**

**What Is New?**

- Our study specifically uncovered miR‐200b‐3p in myocardial infarction and verified its downstream molecular mechanisms by which miR‐200b‐3p carried by mesenchymal stem cells–derived extracellular vesicles relieve myocardial infarction–induced injury via suppressing Bcl‐2–like protein 11 expression.

**What Are the Clinical Implications?**

- To some extent, this research has refreshed the known mechanism of myocardial infarction and supplies another potential to treat myocardial infarction.

Acute myocardial infarction (AMI), commonly referred to as a heart attack, is an event during which blood flow to the heart is reduced or stopped, leading to myocardial necrosis.^1^ AMI occurs commonly as a result of thrombosis in the coronary arteries, as well as coronary embolism, anemia, hypotension, coronary artery dissection, and cocaine use.^2^ The common symptoms of MI are chest pain, angina, and no prior warning signs. Sometimes, mild myocardial infarction (MI) does not produce any symptoms, so it is called a "silent heart attack."^3^ Heart failure is a common complication of MI, and left ventricular systolic dysfunction increases the risk of death.^4^ The heart is also susceptible to oxidative stress, and ischemia changes the defense mechanism against oxygen‐free radicals and produces increased oxygen‐free radicals.^5^ Thus, taking control of oxidative stress and improving cardiac function are promising approaches to managing MI.

Mesenchymal stem cells (MSCs) therapy has achieved great attention and progressed in the therapeutics of MI,^6^, ^7^ and MSCs‐derived extracellular vesicles (MSCs‐EVs) could protect against oxidative stress and improve cardiac function after MI.^8^ In fact, MSCs‐EVs, as a cargo delivery platform, have been expected to treat diseases, including MI.^9^ MSCs‐EVs, such as exosomes, have been widely explored to rescue MI and myocardial ischemia/reperfusion (MI/RI) via microRNA (miRNA) delivery.^10^, ^11^ Differential miRNA profiles are tested in heart failure following MI,^12^ and miR‐200b could prevent adverse myocardial structure and function changes caused by diabetes,^13^ and upregulation of miR‐200b in part could inhibit neuronal apoptosis under hypoxia.^14^ Specifically, miR‐200b‐3p has been examined to be downregulated in diabetic cardiomyopathy, and overexpression of miR‐200b‐3p is cardioprotective to prevent apoptosis.^15^ However, the mechanism of EVs shuttling miR‐200b‐3p in MI has not been completely investigated. BCL2L11 (Bcl‐2–like protein 11) , a target of miR‐200b‐3p predicted online, is a proapoptotic gene in ischemia‐induced heart failure.^16^ On the other hand, BCL2L11 plays adversely in hypoxia/reoxygenation‐conditioned cardiomyocyte injury.^17^ NLR family pyrin domain containing 1 (NLRP1) is an inflammasome whose activation could deteriorate MI/RI,^18^ while deficiency of NLRP1 could fight against cardiac hypertrophy.^19^ We are the first to propose that MSC‐derived EVs carrying miR‐200b‐3p regulate BCL2L11 to inhibit NLRP1 inflammasome activation, and regulate oxidative stress and cardiac function after MI.

## METHODS

The data that support the findings of this study are available from the corresponding author on reasonable request. All animals were treated following the *Guidelines for the Care and Use of Laboratory Animals*, and all experiments were conducted with the guidelines of the ethics committee of Nanfang Hospital, Southern Medical University.

### Culture, Identification, and Transfection of MSCs

MSCs (Shanghai Zhongqiao Xinzhou Biotechnology Co., Ltd.) were maintained in DMEM (10% FBS and 1% penicillin‐streptomycin), and passaged at 1:3 every 2 days.

Flow cytometry was performed to confirm the phenotypic characteristics of MSCs. Fluorescein isothiocyanate–labeled monoclonal antibodies against CD14, CD19, CD73, CD34, CD90, CD45, CD105, and HLA‐DR (1:100, BioLegend) were incubated with MSCs and analyzed using the CyAn ADP analyzer (Beckman Coulter).^20^

MSCs (2×10^5^ cells/mL) were adhered to the wall and transiently transfected with miRNA mimic negative control (NC; miRNA mimic NC, a random sequence miRNA mimic molecule validated to not produce identifiable effects on known miRNA function), or miR‐200b‐3p mimic (miRNA mimic are small, chemically modified double‐stranded RNA molecules designed to specifically bind to and mimic endogenous miRNA molecules and enable miRNA functional analysis by upregulation of miRNA activity) (GenePharma) via Lipofectamine 3000 (Invitrogen) according to the manufacturer’s instructions.^21^

### Isolation and Identification of EVs

MSCs at 90% confluence were incubated in the complete medium containing exosomal‐free FBS for 48 hours. EVs were isolated from the conditioned medium by differential centrifugation. In short, the conditioned medium was subjected to centrifugation at 300*g* (10 minutes) and 2000*g* (20 minutes), and then filtered through a 0.22‐mm filter. An EV pellet was ultracentrifuged at 100 000*g* for 90 minutes, resuspended in PBS, and ultracentrifuged at 100 000*g* for 90 minutes.^22^ The separated EVs were used immediately or stored at −80 °C for subsequent analysis. EVs extracted from MSCs transfected with mimic NC or miR‐200b‐3p mimic were named EVs‐NC or EVs‐miR‐200b‐3p.

Identification of MSC‐EVs was as follows: (1) The morphology of EVs was observed using a transmission electron microscope, and images were captured by a transmission electron microscope (HT7700, 80 kV, Hitachi). (2) The particle size and concentration of EVs were measured by nanoparticle tracking analysis using Nanosight LM10 (Malvern PANalytical) and the data were evaluated by nanoparticle tracking analysis 2.3 software (Malvern PANalytical). (3) The surface markers CD9, CD81, and GRP94 of EVs were measured by Western blotting, and the antibodies used were all purchased from Abcam and diluted at 1:1000.

### Animals

A total of 198 8‐week‐old male c57BL/6 mice (Southern Medical University) were housed (12‐hour light/dark cycle, 60%±5% humidity, 22±3 °C) and allowed to eat and drink freely. All surgical operations were performed under anesthesia to minimize pain for the mice.^23^

### Mouse Model of MI

MI was modeled by ligating the left anterior descending artery. Mice were anesthetized with isoflurane (3% isoflurane for induction and 2% isoflurane for maintenance) and intubated with the 20‐G catheter into the trachea. The catheter was connected to a ventilator (for a mouse with the body weight of 25 g, the tidal volume was set at 225 μL and respiratory rate at 130 times per minute). Also, 100% oxygen was flowed into the ventilator. The thoracic cavity was cut through the left parasternal incision, and the heart was exposed in the 3rd to 4th intercostal space. The pericardium was opened and the left anterior descending artery was ligated with an 8‐0 silk suture (Ethicon). The dissected intercostal space and chest skin were closed with a 6‐0 silk suture (Ethicon). Sham‐operated mice were treated without left anterior descending ligation.^24^

### Treatments of MI Mice

After 24 hours of MI, mice were given an intracardiac injection^24^ of PBS, MSCs‐EVs, EVs‐NC, EVs‐miR‐200b‐3p, MSCs‐EVs+lentivirus‐expressing control short hairpin RNA, MSCs‐EVs+lentivirus‐expressing BCL2L11 short hairpin RNA, MSCs‐EVs+overexpressed‐negative lentivirus, MSCs‐EVs+BCL2L11‐overexpressing lentivirus, EVs‐miR‐200b‐3p+Neg, EVs‐miR‐200b‐3p+BCL2L11, respectively. Mice were injected with EVs (100 μg) or recombinant lentivirus (5×10^7^ viral genome particles per mouse heart). All vectors were provided by Dharmacon. Injection was performed around the infarct area (anterior wall, lateral wall, and apical area) after left anterior descending artery ligation. The average volume at each injection site was about 15 μL. During injection, the needle was located in the ventricular muscle wall, but not in the ventricular cavity. After injection, isoflurane anesthesia was stopped to improve survival.

To assess whether EVs can be absorbed by the myocardium, Dil‐labeled EVs were injected into MI mice and the mice were euthanized 6 hours later. The heart samples were made into frozen 6‐μm sections, which were stained with α‐actinin immunofluorescence and then with 4'‐6‐diamidino‐2‐phenylindole. The internalization of EVs into the myocardium was observed by fluorescence microscope.

### Echocardiography

After 28 days of operation, 6 mice from each group were anesthetized with 1% pentobarbital sodium (40 mg/kg) and fixed on the platform. Left ventricular end‐diastolic diameter (LVEDD) and left ventricular end‐stage systole diameter (LVESD) were continuously measured in 3 cardiac cycles. Left ventricular ejection fraction (LVEF) and left ventricular fractional shortening (LVFS) were calculated.^25^ Heart rate was measured by a programmable tail‐cuff sphygmomanometer (Softron).

### Masson Staining

After detection of cardiac function, the mice were euthanized and myocardial tissues were taken. Myocardial tissues were fixed in 4% formaldehyde overnight and embedded in paraffin. After deparaffinization and rehydratation, 5‐μm sections were prepared. The 5‐μm sections were stained with Masson staining, total collagen deposition was semiquantified by ImageJ system software, and collagen volume fraction was calculated. At least 5 fields were randomly selected for each section.^26^

### Hematoxylin‐Eosin Staining

Paraffin sections of myocardial tissues (5 μm) were soaked in xylene, immersed in gradient ethanol, stained with hematoxylin‐eosin, and then immersed in gradient ethanol. After permeabilization in xylene, the sections were sealed with neutral resin and observed under an optical microscope.^27^

### Terminal Deoxynucleotidal Transferase–Mediated Biotin–Deoxyuridine Triphosphate Nick‐End Labeling Staining

According to the manufacturer's instructions, a fluorescein in situ cell death detection kit (Roche) was used to detect apoptotic cardiomyocytes in the paraffin sections of myocardial tissues. The sections were counteracted with 4'‐6‐diamidino‐2‐phenylindole. The apoptotic cells were analyzed by fluorescence microscope (Nikon, Eclipse TI‐SR), and TUNEL (terminal deoxynucleotidal transferase–mediated biotin–deoxyuridine triphosphate nick‐end labeling)–positive cells were counted by Image‐Pro Plus 6.0 software (Media Cybernetics Inc).^28^

### 2,3,5‐Triphenyltetrzoliumchloride Staining

The left anterior descending branch of the optical artery was re‐ligated with the sutures reserved in the original position of the mouse heart ligation, and 1 mL of Evans blue dye was retrogradely perfused through the aortic arch with a 1‐mL syringe. Outside the anterior wall of the left ventricle, other areas of the heart were stained blue. The heart was immediately immersed in precooled saline and frozen at −20 °C. Afterwards, the heart was cross‐sectioned below the ligation site, placed in 2% 2,3,5‐triphenyltetrzoliumchloride (TTC) solution and fixed in 10% neutral formalin. The blue area was the distal myocardium, the nonblue area (red area and uncolored white area) was the area at risk, and the white area was the area of infarction. The area of infarction/area at risk was calculated by Image‐Pro Plus software. Six mice were selected from each group.

### Detection of Oxidative Stress

The heart tissue in the border zone of the infarct area was homogenized in RIPA lysis buffer and centrifuged at 12 000*g* for 10 minutes. The supernatant was collected to measure malondialdehyde, superoxide dismutase (SOD) activity, and reactive oxygen species (ROS) as the kits’ instruction recommended (NanJing JianCheng Bioengineering Institute).^29^

### Reverse Transcription Quantitative Polymerase Chain Reaction

Total RNA was separated with Trizol reagent and reverse‐transcribed into cDNA using the miRcute Plus miRNA First‐Strand cDNA kit (Tiangen) for miRNA or FastKing RT kit (Tiangen) for mRNA. miRNA was quantified using the miRcute Plus miRNA qPCR kit (Tiangen), and mRNA was quantified using the SuperReal PreMix Plus kit (Tiangen). Gene expression was normalized to U6 or GAPDH and calculated by the 2^−ΔΔCt^ method. See Table S1 for polymerase chain reaction (PCR) sequences.

### Western Blot Assay

Total protein from myocardial tissues was separated using a lysis buffer containing protease inhibitor. After centrifugation at 12 000 *g*, the supernatant was separated on a 10% sodium dodecyl sulphate‐polyacrylamide gel, transferred to a nitrocellulose membrane, and combined with primary antibodies against BCL2L11 (1:1000, Abcam) and NLRP1 (1:1000; Abcam). Anti‐GAPDH (1:10 000, Abcam) was the internal control. Then, the membrane was mixed with appropriate secondary antibody and detected by an enhanced chemiluminescence kit (Amersham Pharmacia). The images were captured by the image analysis system (Bio‐Rad) and analyzed with Quantity One software.^30^

### Dual Luciferase Reporter Gene Assay

BCL2L11 3'‐UTR was amplified by PCR. Mutant 3'‐UTR was generated by QuikChange II XL site‐directed mutagenesis kit (Stratagene). The wild‐type BCL2L11 3'‐UTR (BCL2L11‐WT, UCUAUGAAUUGUAGAAGUAUUC) and the mutant‐type BCL2L11 3'‐UTR (BCL2L11 mutant, UCUAUGAAUUGUAGACAGCGGC) were cloned into the downstream of the coding region of the luciferase gene. The constructed vector was co‐transfected with miR‐200b‐3p mimic or mimic NC into 293T cells through Lipofectamine 3000 (Invitrogen). The luciferase report analysis system (Promega) was utilized to measure the luciferase activity.^31^

### RNA Pull‐Down Assay

Cells were transfected with 50 mmol/L of biotinylated miRNA mimic or the vector control. The cells were incubated in lysis buffer (Invitrogen), centrifuged, and combined with streptavidin sepharose beads (Invitrogen). The beads were boiled in sodium dodecyl sulfate and subjected to reverse transcription quantitative PCR detection.^31^

### Co‐Immunoprecipitation Assay

Cells were lysed by radioimmunoprecipitation assay (Solarbio) containing protease inhibitor (Roche Diagnostics GmbH). The primary antibodies anti‐BCL2L11 (1:50, Abcam) and anti‐NLRP1 (1:100; Abcam) were incubated with protein A/G‐Sepharose beads (Santa Cruz Biotechnology), and the precipitate was analyzed by Western blot.^20^

### Statistical Analysis

Data were presented as the mean±SD. The normality of data distribution was analyzed using the Kolmogorov‐Smirnov test, and the homogeneity of variances was assessed using the Levene test. Data were analyzed using SPSS version 21.0 (IBM) and GraphPad Prism version 7 (GraphPad Software). Difference analysis was conducted through *t*‐test and ANOVA followed by post hoc Tukey test. *P*<0.05 represented statistical significance.^32^

## RESULTS

### Identification of MSCs and EVs

**Figure 1:**
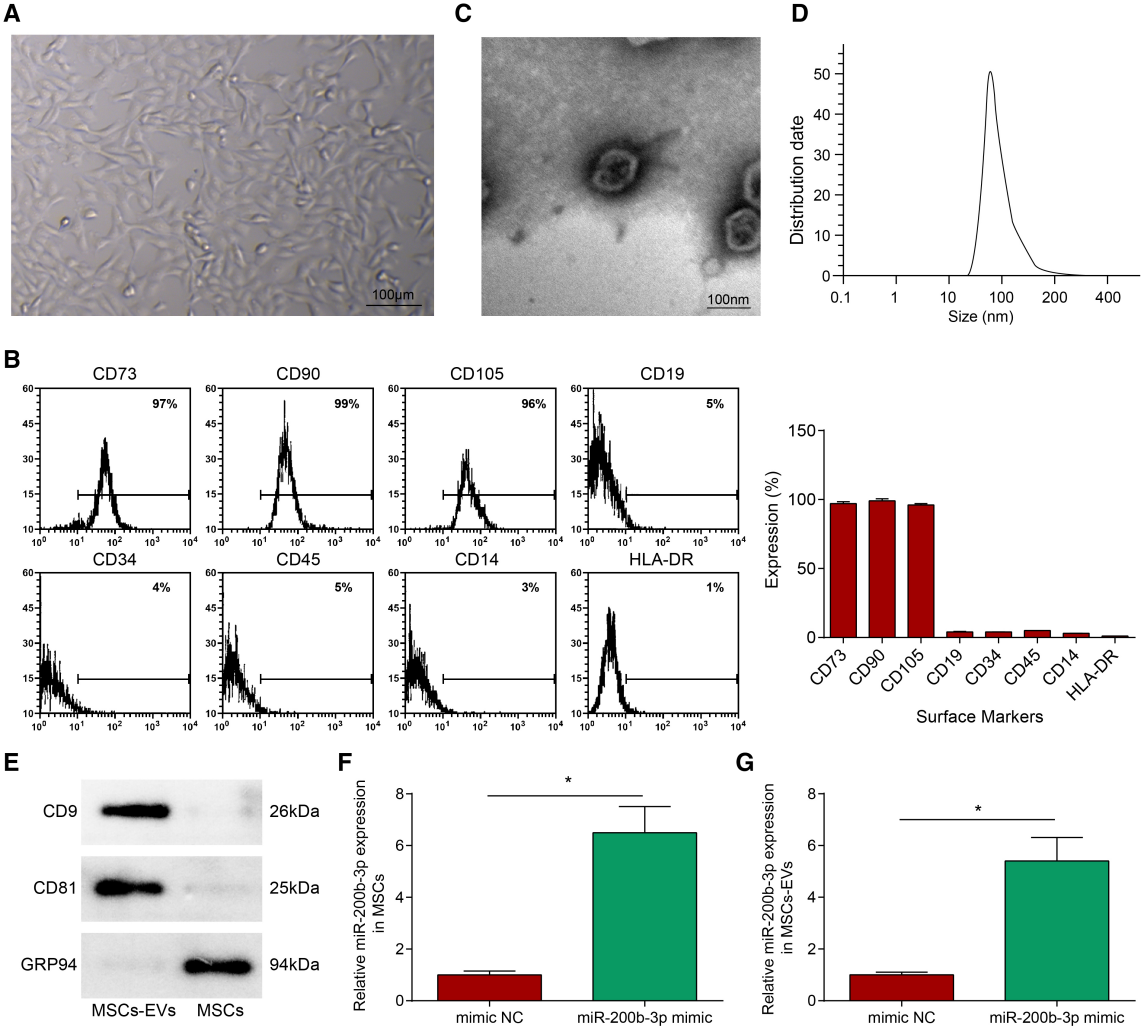
Identification of mesenchymal stem cells (MSCs) and extracellular vesicles (EVs). **A**, Observation of MSCs. **B**, Flow cytometry detection of MSCs surface markers. **C**, Transmission electron microscope observation of EVs. **D**, Nanoparticle tracking analysis of EVs. **E**, Western blot detection of EVs protein markers in MSCs debris and MSCs‐EVs. **F** and **G**, miR‐200b‐3p expression in MSCs and MSCs‐EVs. N=3. Data are shown as mean±SD and evaluated by *t*‐test. *P<0.05. NC indicates negative control.

MSCs isolated from the umbilical cord showed a spindle‐like fibrous morphology (Figure 1A). Flow cytometry (Figure 1B) showed that the isolated cells were in good homogeneity and high purity; CD73 (97%), CD90 (99%), and CD105 (96%) were highly expressed, while CD19 (5%), CD34 (4%), CD45 (5%), CD14 (3%), and HLA‐DR (1%) were lowly expressed. Thus, the isolated cells were MSCs.

In addition, EVs were isolated from the conditioned medium of MSCs. Under the transmission electron microscope, MSCs‐EVs were cup‐shaped or saccular (Figure 1C); nanoparticle tracking analysis showed that the diameter of MSCs‐EVs ranged from ≈30 nm to 150 nm (Figure 1D). Western blot analysis revealed that CD81 and CD9 were evidently expressed on EVs, but not GRP94 (Figure 1E). Thus, EVs from MSCs were successfully isolated.

Reverse transcription quantitative PCR analyzed miR‐200b‐3p level in MSCs and MSCs‐EVs, confirming that miR‐200b‐3p mimic increased miR‐200b‐3p level in MSCs and MSCs‐EVs compared with mimic NC (Figure 1F and 1G), indicating successful transfection.

### MSCs‐EVs Loaded With miR‐200b‐3p Effectively Improve Myocardial Pathological Changes and Reduce Cardiomyocyte Apoptosis in MI Mice

**Figure 2:**
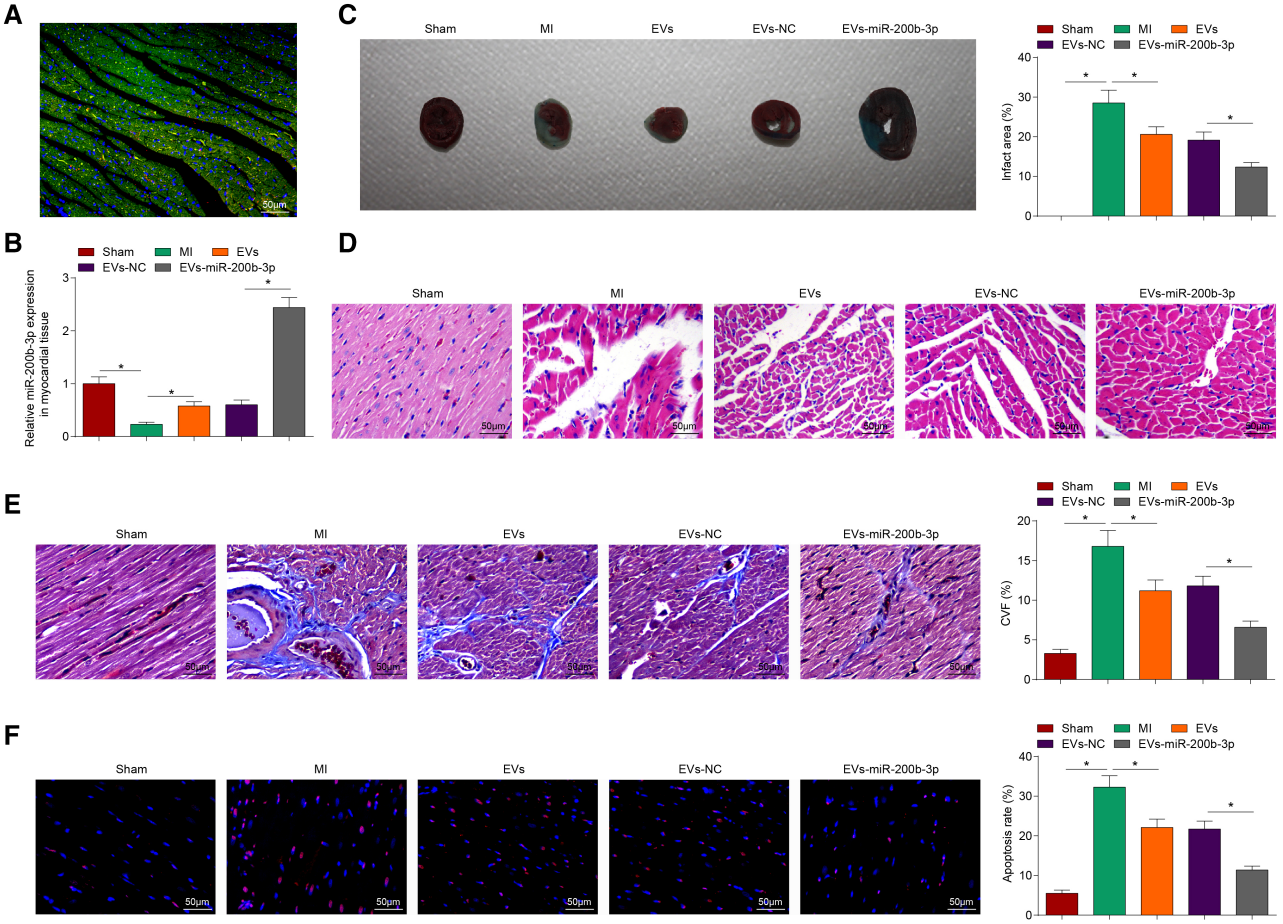
Mesenchymal stem cells (MSCs)–extracellular vesicles (EVs) effectively reduce pathological damage and cardiomyocyte apoptosis in myocardial infarction (MI) mice through miR‐200b‐3p. **A**, Dil‐labeled EVs were injected into the infarcted heart of mice for 6 hours. **B**, Reverse transcription quantitative polymerase chain reaction detection of miR‐200b‐3p expression. **C**, Infarction area of mice observed by 2,3,5‐triphenyltetrzoliumchloride staining. **D**, Pathological characteristics of mice observed by hematoxylin‐eosin staining. **E**, Collagen volume fraction detected by Masson staining. **F**, Cardiomyocyte apoptosis rate detected by terminal deoxynucleotidal transferase–mediated biotin–deoxyuridine triphosphate nick‐end labeling) staining. n=6 each. Data are shown as mean±SD and evaluated by ANOVA. *P<0.05. NC indicates negative control.

DiL‐labeled EVs were injected into the myocardium to observe whether EVs can be internalized by cardiomyocytes. After 6 hours, immunofluorescence staining was performed, finding that Dil‐labeled EVs co‐localized with cardiomyocytes, indicating that cardiomyocytes can effectively take up EVs (Figure 2A). Subsequently, reverse transcription quantitative PCR found that (Figure 2B) the decreased miR‐200b‐3p level in MI mice was restored after EVs treatment, and EVs‐miR‐200b‐3p had a better effect on elevating miR‐200b‐3p expression in MI mice. It was seen in TTC staining that there was no loss of myocardial tissue in sham‐operated mice, and obvious infarct area was measured in MI mice; EVs treatment or EVs‐miR‐200b‐3p treatment reduced the infarct size of MI mice, and EVs‐miR‐200b‐3p treatment was more effective (Figure 2C). Hematoxylin‐eosin staining observed the myocardial pathological changes of mice and the outcomes found that myocardial structure disorder, interstitial hemorrhage, leucocyte infiltration, myocardial swelling and intercellular space widening, and focal degeneration of myofibrils were visible in MI mice (Figure 2D). After EVs or EVs‐miR‐200b‐3p treatment, myocardial histopathological abnormalities were improved in MI mice, and EVs‐miR‐200b‐3p treatment was more effective. Masson staining was performed to observe myocardial fibrosis, showing that increased fibrotic areas in the myocardial tissue of MI mice was reduced after injection of EVs or EVs‐miR‐200b‐3p, and the effect of EVs‐miR‐200b‐3p was better (Figure 2E). TUNEL staining applied to detect cardiomyocyte apoptosis reported that increased TUNEL‐positive cells in the myocardial tissue of MI mice was decreased after injection of EVs or EVs‐miR‐200b‐3p, and EVs‐miR‐200b‐3p had a greater effect (Figure 2F).

### MSCs‐EVs Carrying miR‐200b‐3p Reduce Myocardial Inflammatory Response and Oxidative Stress and Improve Cardiac Function in MI Mice

**Figure 3:**
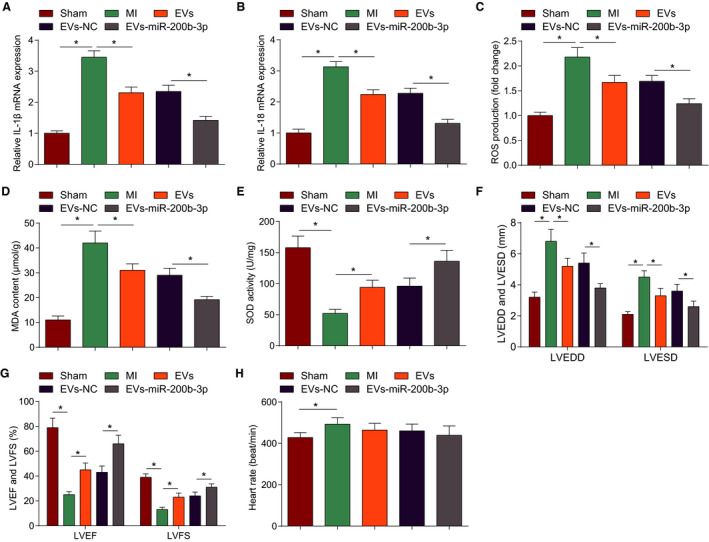
Extracellular vesicles (EVs) can effectively inhibit inflammatory response and oxidative stress and improve cardiac function in myocardial infarction (MI) mice through miR‐200b‐3p. **A** and **B**, Interleukin (IL)‐1β and IL‐18 levels in myocardial tissues of MI mice. **C**, Reactive oxygen species (ROS) content in myocardial tissues of mice. **D** and **E**, Malondialdehyde (MDA) content and superoxide dismutase (SOD) activity in myocardial tissues of mice. **F**, Left ventricular end‐diastolic diameter (LVEDD) and left ventricular end‐stage systole diameter (LVESD) in mice. **G**, Left ventricular ejection fraction (LVEF) and left ventricular fractional shortening (LVFS) in mice. **H**, Heart rate in mice. n=6 each. Data are shown as mean±SD and evaluated by ANOVA. *P<0.05. NC indicates negative control.

Reverse transcription quantitative PCR analyzed inflammatory factors, and the results suggested that interleukin (IL)‐1β and IL‐18 levels were increased in the myocardial tissue of MI mice but could be suppressed by EVs or EVs‐miR‐200b‐3p treatment. Moreover, the EVs‐miR‐200b‐3p–mediated decrease of IL‐1β and IL‐18 levels was more obvious than EVs (Figure 3A and 3B).

We then tested myocardial oxidative stress injury and cardiac function and observed that MI mice had raised malondialdehyde and ROS contents and impaired SOD activity (Figure 3C through 3E), as well as increased LVEDD, LVESD, and heart rate, and decreased LVEF and LVFS (Figure 3F through 3H), indicating that oxidative stress was enhanced and cardiac function was destroyed after MI. After injection of EVs or EVs‐miR‐200b‐3p in MI mice, malondialdehyde and ROS contents were suppressed and SOD activity was strengthened, LVEDD, and LVESD were reduced, LVEF and LVFS were enhanced; the improvement effect of EVs‐miR‐200b‐3p treatment was better than EVs treatment. In summary, MSCs‐EVs can deliver miR‐200b‐3p to effectively inhibit myocardial inflammation and oxidative stress damage and improve cardiac function in MI.

### BCL2L11 is Targeted by miR‐200b‐3p and Binds to NLRP1

**Figure 4:**
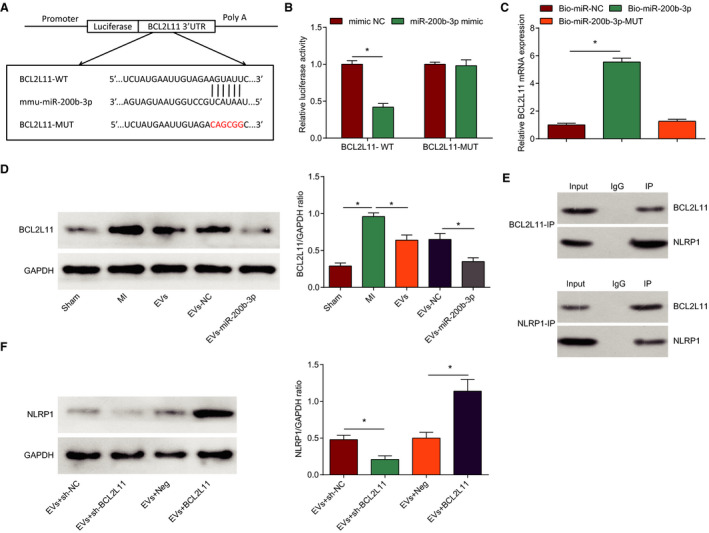
BCL2L11 (Bcl‐2–like protein 11) is targeted by miR‐200b‐3p and binds to NLR family pyrin domain containing 1 (NLRP1). **A**, Target relationship prediction diagram. **B**, Dual luciferase experimental analysis results. **C**, RNA pull‐down experimental results. **D**, Western blot detection of BCL2L11 expression. **E**, Co‐immunoprecipitation (IP) experimental results. **F**, Western blot detection of NLRP1 expression. N=3; n=6 each. Data are shown as mean±SD and evaluated by *t*‐test or ANOVA. EVs indicate extracellular vesicles; MI, myocardial infarction; and NC, negative control. *P<0.05.

The bioinformatics software starBase was used to identify potential targets of miR‐200b‐3p, and BCL2L11 was selected because of its role in myocardial injury.^33^, ^34^, ^35^ To confirm the interaction between miR‐200b‐3p and BCL2L11, a luciferase reporter vector containing BCL2L11 3'UTR WT or mutant was constructed (Figure 4A). Overexpression of miR‐200b‐3p suppressed the luciferase activity of BCL2L11‐WT but not BCL2L11 mutant in 293T cells (Figure 4B).

We also conducted RNA pull‐down experiment and observed that biotinylated miR‐200b‐3p enriched the expression of BCL2L11, which confirmed the interaction between miR‐200b‐3p and BCL2L11 (Figure 4C). Subsequently, Western blotting analyzed BCL2L11 expression and measured an increase of BCL2L11 expression in the myocardial tissue of MI mice more than sham‐operated mice. In MI mice, expression analysis exhibited that after EVs or EVs‐miR‐200b‐3p treatment, BCL2L11 expression was suppressed. EVs‐miR‐200b‐3p treatment was more effective for decreasing BCL2L11 expression (Figure 4D).

NLRP1 inflammasome activation promotes MI/RI^18^ and NLRP1 is upregulated in atherosclerosis.^36^ The interaction between BCL2L11 and NLRP1 was confirmed by co‐immunoprecipitation assay (Figure 4E): BCL2L11 interacted with NLRP1 in cells. We examined NLRP1 expression changed in mouse myocardial tissues (Figure 4F) and found that NLRP1 expression was increased or decreased according to the change of BCL2L11 expression. Overall, BCL2L11 was targeted by miR‐200b‐3p and bound to NLRP1.

### Downregulating BCL2L11 Enhances MSCs‐EVs–Mediated Effect on Improving Myocardial Pathology and Reducing Cardiomyocyte Apoptosis in MI Mice

**Figure 5:**
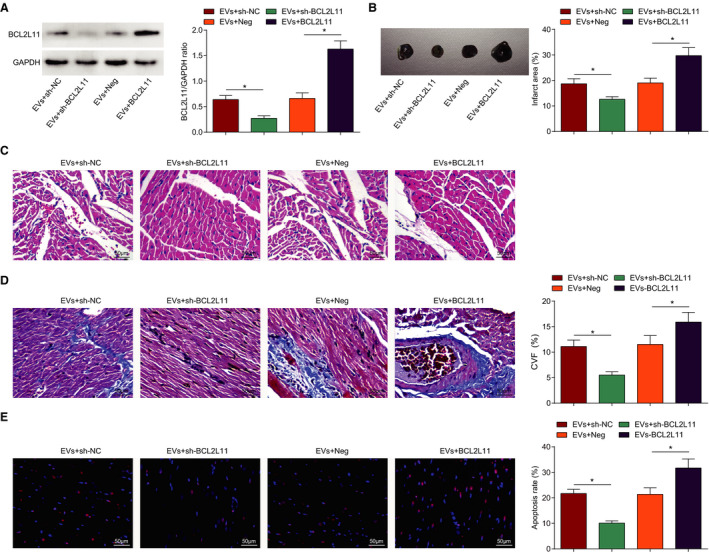
Downregulating BCL2L11 (Bcl‐2–like protein 11) enhances mesenchymal stem cells–extracellular vesicles (EVs)–mediated effect on improving myocardial pathology and reducing cardiomyocyte apoptosis in myocardial infarction mice. **A**, Western blot detection of BCL2L11 expression. **B**, Infarction area of mice observed by 2,3,5‐triphenyltetrzoliumchloride staining. **C**, Pathological characteristics of mice observed by hematoxylin‐eosin (HE) staining. **D**, Collagen volume fraction (CVF) detected by Masson staining. **E**, Cardiomyocyte apoptosis rate detected by terminal deoxynucleotidal transferase–mediated biotin–deoxyuridine triphosphate nick‐end labeling staining. n=6 each. Data are shown as mean±SD and evaluated by ANOVA. *P<0.05. NC indicates negative control.

Our study confirmed the inhibitory effect of EVs treatment on reducing BCL2L11 expression in the myocardial tissue of MI mice; thus, the role of BCL2L11 was explored in EVs‐treated MI mice. First, MI mice were co‐injected with EVs and BCL2L11‐related vector, and Western blot detected that (Figure 5A) BCL2L11 expression was reduced by MSCs‐EVs+lentivirus‐expressing BCL2L11 short hairpin RNA treatment relative to MSCs‐EVs+lentivirus‐expressing control short hairpin RNA treatment, while BCL2L11 expression was increased by MSCs‐EVs+BCL2L11‐overexpressing lentivirus treatment than MSCs‐EVs+overexpressed‐negative lentivirus treatment. Histological staining indicated that sh‐BCL2L11 enhanced the protective effects of EVs on reducing infarction area, improving myocardial damage, and reducing fibrotic areas and TUNEL‐positive cells, while BCL2L11 overexpression caused opposite consequences (Figure 5B through 5E).

### BCL2L11 Silencing Enhances EVs‐Mediated Reduction in Myocardial Inflammatory Response, Oxidative Stress, and Improvement of Cardiac Function in MI Mice

**Figure 6:**
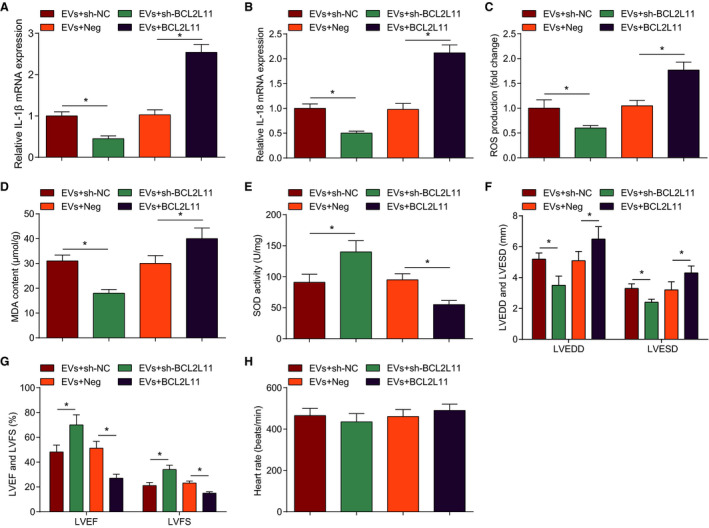
BCL2L11 (Bcl‐2–like protein 11) silencing enhances extracellular vesicles (EVs)–mediated reduction in myocardial inflammatory response, oxidative stress, and improvement of cardiac function in myocardial infarction (MI) mice. **A** and **B**, Interleukin (IL)‐1β and IL‐18 levels in myocardial tissues of mice. **C**, Reactive oxygen species (ROS) content in myocardial tissues of mice. **D** and **E**, Malondialdehyde (MDA) content and superoxide dismutase (SOD) activity in myocardial tissues of mice. **F**, Left ventricular end‐diastolic diameter (LVEDD) and left ventricular end‐stage systole diameter (LVESD) in mice. **G**, Left ventricular ejection fraction (LVEF) and left ventricular fractional shortening (LVFS) in mice. **H**, Heart rate in mice. n=6 each. Data are shown as mean±SD and evaluated by ANOVA. *P<0.05. NC indicates negative control.

Detection of inflammatory response (Figure 6A and 6B), oxidative stress (Figure 6C through 6E), and cardiac function (Figure 6F through 6H) manifested that sh‐BCL2L11 promoted the role of EVs in decreasing IL‐1β and IL‐18 levels, suppressing malondialdehyde and ROS contents, and improving SOD activity, as well as reducing LVEDD and LVESD and increasing LVEF and LVFS; while the effect of EVs was reversed by BCL2L11 upregulation in MI mice. In conclusion, downregulating BCL2L11 enhanced MSCs‐EVs–mediated improvement of MI in mice.

### Upregulation of BCL2L11 Negates the Role of EVs‐miR‐200b‐3p in MI Mice

**Figure 7:**
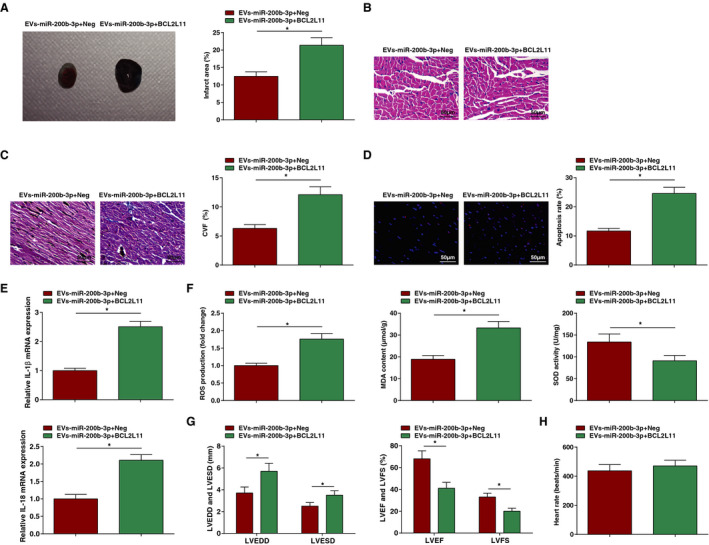
Upregulation of BCL2L11 (Bcl‐2–like protein 11) negates the role of miR‐200b‐3p–modified mesenchymal stem cells (MSCs)–extracellular vesicles (EVs) in myocardial infarction (MI) mice. **A**, Infarction area of mice observed by 2,3,5‐triphenyltetrzoliumchloride staining. **B**, Pathological characteristics of mice observed by hematoxylin‐eosin staining. **C**, Collagen volume fraction (CVF) detected by Masson staining. **D**, Cardiomyocyte apoptosis rate detected by terminal deoxynucleotidal transferase–mediated biotin–deoxyuridine triphosphate nick‐end labeling staining. **E**, Interleukin (IL)‐1β and IL‐18 levels in mice. **F**, Malondialdehyde (MDA) content, superoxide dismutase (SOD) activity, and reactive oxygen species (ROS) content in myocardial tissues of mice. **G**, Left ventricular end‐diastolic diameter (LVEDD), left ventricular end‐stage systole diameter (LVESD), left ventricular ejection fraction (LVEF), and left ventricular fractional shortening (LVFS) in mice. **H**, Heart rate in mice. n=6 each. Data are shown as mean±SD and evaluated by *t*‐test. *P<0.05.

For exploring the effect of EVs carrying miR‐200b‐3p regulating BCL2L11 on MI, MI mice were injected with EVs‐miR‐200b‐3p+BCL2L11 to specifically explore the molecular mechanism. Through a series of assays, we found that in MI mice treated with EVs‐miR‐200b‐3p+BCL2L11, the infarction area was widened (Figure 7A), myocardial pathological damage was worsened (Figure 7B), fibrotic area was increased (Figure 7C), TUNEL‐positive apoptotic cells were increased (Figure 7D), inflammatory response (Figure 7E) and oxidative stress (Figure 7F) were promoted, and cardiac function (Figure 7G and 7H) was impaired relative to those treated with EVs‐miR‐200b‐3p+Neg. In summary, upregulation of BCL2L11 negated the role of EVs‐miR‐200b‐3p in MI mice.

## DISCUSSION

**Figure 8:**
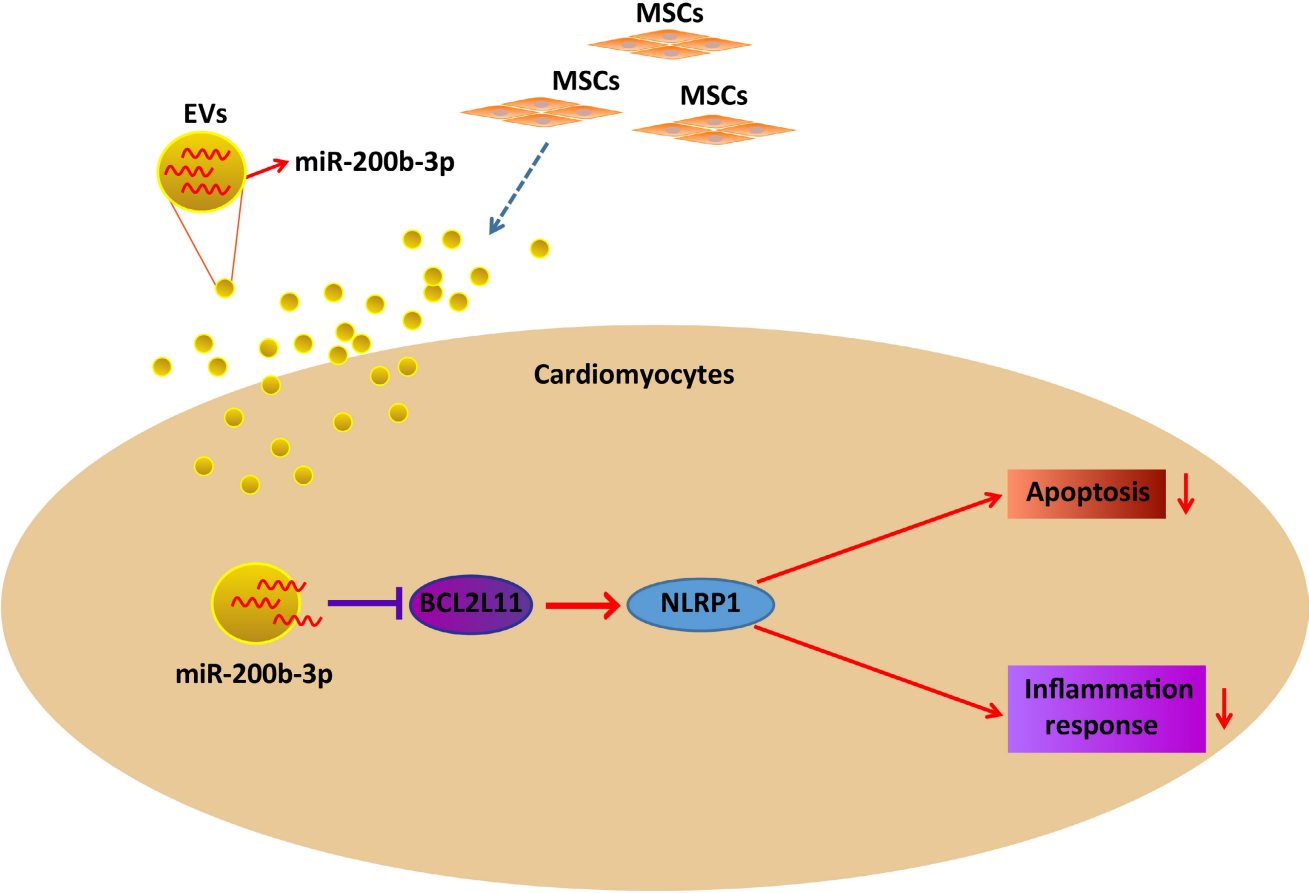
Molecular mechanism diagram. Mesenchymal stem cells (MSCs)–extracellular vesicles (EVs) secrete miR‐200b‐3p, thereby inhibiting the transcription of BCL2L11 (Bcl‐2–like protein 11) , further inhibiting the activation of NLR family pyrin domain containing 1 (NLRP1) inflammasomes, thus treating cardiomyocyte apoptosis and myocardial inflammatory response in myocardial infarction mice.

AMI is the most serious event after coronary artery disease, occupying a considerable share of global health.^37^ Much effort has been made to treat MI and our study in part provided a potential strategy based on MSCs‐EVs delivery of miR‐200b‐3p. In detail, miR‐200b‐3p from MSCs‐EVs could relieve MI via reducing oxidative stress and repairing cardiac function through downregulating BCL2L11 and inactivating NLRP1 (Figure 8).

Initially, our experimental data highlighted that injection of MSCs‐EVs into the heart of mice with MI could attenuate the infarction area and pathological injury, reduce collagen volume fraction value and apoptotic rate, restore cardiac function (decreased heart rate, LVEDD, LVESD, and left ventricular end‐diastolic pressure, and increased LVEF, LVFS, and LVSP), and suppress oxidative stress (reduced malondialdehyde and ROS and enhanced SOD activity). The biological characteristics of MSCs depend on their endocrine or paracrine substances, and exosomes play an important biological role in the paracrine mechanism.^38^, ^39^, ^40^ As suggested previously, the treatment effects of MSCs to reduce infarction and improve cardiac function for MI are mainly attributed to the paracrine effect.^41^, ^42^ It is worth noting that MSCs‐derived exosomes could limit the secretion of ROS and suppress apoptosis of cardiomyocytes in vitro, as well as narrow infarct size and improve cardiac function in vivo.^43^ Also, application of human umbilical cord MSCs–secreted exosomes to AMI rats could reduce malondialdehyde content and ameliorate myocardial injury.^44^ More significantly, the therapeutic impact of MSCs‐derived exosomes has been suggested in MI/RI, as reflected by reduced oxidative stress, enhanced cardiomyocyte viability, and prevented adverse remodeling.^45^ Reported recently, a paper has illustrated that hypoxia‐conditioned MSCs‐derived exosomes could assist cardiac repair through transfer of miR‐125b‐5p to reduce cardiomyocyte death in MI.^46^ In a previous observational study, it was established that treatment of MSCs‐derived exosomes confers an apoptosis reduction of cardiomyocytes in vitro and an improvement of cardiac function in vivo through transportation of miR‐338 in the context of MI.^47^ Also, in porcine ischemia‐reperfusion, MSC‐EVs reduce the infarct range and improve heart function^45^ and the same result can be obtained in the mouse model.^48^ Indeed, ncRNAs often interact with proteins to play a role in the process of EVs delivery,^49^ and ncRNAs are involved in the MSCs‐EVs delivery in the treatment of MI. For instance, MSCs‐EVs delivery of miR‐338 could suppress the apoptosis of cardiomyocytes in MI,^47^ and that of miR‐144‐3p could prevent the mobilization of endothelial progenitor cells after MI.^50^ In total, the strategy of MSCs‐EVs (including exosomes) has validated its therapeutics in MI.

Concerning miR‐200b‐3p, it was downregulated in MI and overexpression of miR‐200b‐3p could further strengthen the protective effects of MSCs‐EVs in disease. In fact, a study has reported that miR‐200b‐3p demonstrates a low expression level in diabetic cardiomyopathy and raising miR‐200b‐3p expression restrains the apoptotic phenotype of cardiomyocytes, as well as rehabilitates cardiac function and attenuates myocardial pathology.^15^ Of note, hypoxia‐responsive miR‐200b‐3p is pronouncedly downregulated in patients with coronary artery disease, showing an association with cardiac complications and the pathophysiological state of patients.^51^ In hypoxia‐induced neuronal injury, upregulating miR‐200 family (including miR‐200b) has a considerable impact on suppressing neuronal apoptosis.^14^ Accordingly, a study has examined the decreased level of miR‐200b in the process of cardiac fibrosis and upregulation of miR‐200b represses the fibrotic process.^52^ Our study findings and these reports all support the cardioprotective role of miR‐200b‐3p.

BCL2L11, a confirmed target of miR‐200b‐3p, was shown to be involved in the process of miR‐200b‐3p–mediating MI. In detail, we investigated that downregulating BCL2L11 enhanced the effects of miR‐200b‐3p–modified MSCs‐EVs on MI. In the course of MI/RI, knocking down BCL2L11 relieves hypoxia/reoxygenation‐induced injury for cardiomyocytes via improving cell viability and inhibiting apoptosis.^17^ Additionally, miR‐101a–targeted downregulation of BCL2L11 is partially attributable to the relives of oxidative stress and cardiomycyte apoptosis.^53^ Intriguingly, silencing of BCL2L11 at least contributes to miR‐19b‐1 overexpression–mediated apoptosis reduction and heart failure amelioration.^16^ In addition, another article has shown that inhibition of BCL2L11 reverses cardiac dysfunction and could lessen the apoptotic activity of cardiomyocytes in mice with MI/RI.^35^ Experimentally, miR‐24–mediated attenuation of AMI has been validated to be accredited to repression of BCL2L11.^54^ Overall, the role of BCL2L11 in our study was similar to other data in the field. Finally, we observed a binding relationship between BCL2L11 and NLRP1, and detected the changes of NLRP1 expression during EVs‐miR‐200b‐3p/BCL2L11–treating MI. In fact, NLRP1 activation is one of the contributors of MI/RI.^18^

## CONCLUSIONS

Our study specifically studied miR‐200b‐3p in MI and verified its downstream molecular mechanisms by which miR‐200b‐3p carried by MSCs‐EVs relieves MI‐induced injury via suppressing BCL2L11 expression. To some extent, this research has refreshed the known mechanism of MI and supplies another potential to treat MI. Whether there may be lncRNA or transcription protein upstream of miR‐200b‐3p and a related unknown signaling pathway downstream of the BCL2L11/NLRP3 axis is the direction for our follow‐up research. The infarct size of sham is 0, so we did not consider the use of EVs for treatment, which is a limitation of the study.

## Sources of Funding

This work was supported by the Presidential Foundation of Nanfang Hospital, China (grant No. 2019B005).

## Disclosures

None.

## Supporting information

Table S1
