# Supplementary material for: Protective Effects of MicroRNA‐200b‐3p Encapsulated by Mesenchymal Stem Cells–Secreted Extracellular Vesicles in Myocardial Infarction Via Regulating BCL2L11
Source: J Am Heart Assoc. 2022 Jun 14;11(12):e024330. doi: 10.1161/JAHA.121.024330 (PMC9238663; doi:10.1161/JAHA.121.024330)
Supplement: Supplementary file 1 — Table S1 [file JAH3-11-e024330-s001.pdf]

SUPPLEMENTAL MATERIAL

Table S1. Primers.

Target genes	Primer sequences (5’ to 3’)
miR-200b-3p	Forward: TAATACTGCCTGGTAATGATGA
	Reverse: the reverse primer for miR-200b-3p was universal (included in the miRcute Plus miRNA qPCR kit)
U6	Forward: GGAACGATACAGAGAAGATTAGC
	Reverse: TGGAACGCTTCACGAATTTGCG
BCL2L11	Forward: TCGTTCGATCGGCGCAACA
	Reverse: TGCCGGGCTCCTGTCTTG
IL-1β	Forward: CACCTCACAAGCAGAGCACAAG
	Reverse: GCATTAGAAACAGTCCAGCCCATAC
IL-18	Forward: ACGTGTTCCAGGACACAACA
	Reverse: GGCGCATGTGTGCTAATCAT
GAPDH	Forward: TGGTGAAGCAGGCATCTGAG
	Reverse: TGTTGAAGTCGCAGGAGACAAC

Abbreviations: miR-200b-3p, microRNA-200b-3p; BCL2L11, Bcl-2-like protein 11; IL-1β, interleukin-1β; IL-18, interleukin-18; GAPDH, glyceraldehyde-3-phosphate dehydrogenase.
